# Fahr Syndrome Secondary to Pseudohypoparathyroidism

**DOI:** 10.1210/jcemcr/luad147

**Published:** 2023-12-01

**Authors:** Sharon W Shu, Sakshi Sharma, Qasim Z Iqbal, Karina G Romo

**Affiliations:** Department of Internal Medicine, Ochsner Medical Center, New Orleans, LA 70121, USA; University of Queensland-Ochsner Clinical School, Brisbane, QLD 4072, Australia; Department of Internal Medicine, Ochsner Medical Center, New Orleans, LA 70121, USA; Department of Internal Medicine, Ochsner Medical Center, New Orleans, LA 70121, USA; Endocrinology Division, Indiana University School of Medicine, Indianapolis, IN 46202, USA; Department of Internal Medicine, Ochsner Medical Center, New Orleans, LA 70121, USA; Department of Endocrinology, Cedars-Sinai Medical Center, Los Angeles, CA 90048, USA

**Keywords:** Fahr syndrome, pseudohypoparathyroidism, hypocalcemia, hypokalemia

## Abstract

Fahr syndrome is a rare neurologic disorder, usually affecting young and middle-aged adults, that can present with symptoms ranging from extrapyramidal to neuropsychiatric abnormalities. Pseudohypoparathyroidism (PHP), characterized by parathyroid hormone (PTH)-resistance or PTH-unresponsiveness at target organs, is associated with Fahr syndrome and typically presents with hypocalcemia. The following case presents a 39-year-old-woman with PHP complicated by symptomatic hypocalcemia, hypokalemia, and movement disturbances, who had computed tomography imaging showing basal ganglia calcifications consistent with Fahr syndrome. She initially presented with headache and was hospitalized for hypertensive emergency and severe hypocalcemia. Examination, including the neurologic examination, was unrevealing aside from hypertension and central adiposity. Laboratory tests were consistent with PHP, showing hypocalcemia with elevated PTH, and negative for hyperaldosteronism. Management of hypocalcemia consisted of intravenous calcium infusion, oral calcium carbonate, oral vitamin D3, and oral calcitriol. Patients with severe hypocalcemia and elevated PTH who present with new neurological symptoms despite normal general neurologic examination may warrant consideration for brain imaging to evaluate for Fahr syndrome. Further investigations are necessary to determine the prevalence of Fahr syndrome and hypokalemia in patients with PHP, explore if these findings are significantly associated with PHP-1b subtype, and ultimately inform potential new screening pathways for these patients.

## Introduction

Fahr syndrome is a rare neurologic disorder, usually affecting young and middle-aged adults, that can present with an array of symptoms ranging from extrapyramidal to neuropsychiatric abnormalities [1]. It is characterized by calcium carbonate or calcium phosphate deposits, typically found in the basal ganglia, but also reported in the cerebral cortex, thalamus, hippocampus, and dentate nucleus [2]. Common clinical manifestations of bilateral basal ganglia calcifications include parkinsonism, depression, and cognitive impairment. Unlike Fahr disease, which is most often inherited in an autosomal dominant pattern and defined as primary basal ganglia calcifications without known etiology, Fahr syndrome is secondary to a known etiology. Although the exact pathophysiology of this disorder is unknown, it is associated with endocrine disorders, mitochondrial myopathies, dermatological conditions, and infectious diseases. Of these, parathyroid hormone (PTH) abnormalities are the most commonly implicated cause, including idiopathic hypoparathyroidism, secondary hypoparathyroidism, pseudohypoparathyroidism (PHP), pseudo-pseudohypoparathyroidism, and hyperparathyroidism [3].

PHP is a rare, heterogeneous group of disorders in which target organs demonstrate resistance or unresponsiveness to PTH due to genetic mutations. Typically, it is classified as Type 1, which has a blunted cyclic AMP (cAMP) response, or the much rarer Type 2, which has a normal cAMP response. PHP Type 1 is then further subclassified as Type 1a, 1b, or 1c. PHP-1a and PHP-1c may both express a phenotype of Albright hereditary osteodystrophy (AHO), such as short stature, obesity, below average intelligence, brachydactyly, or shortened metatarsals—they are differentiated by Gs alpha function, which is reduced in PHP-1a. In contrast, PHP-1b is the only subtype without clinical features of AHO; this phenotype is attributed to isolated renal PTH-resistance with normal PTH response in bone [4].

As a group, PHP is biochemically characterized by hypocalcemia, hyperphosphatemia, and paradoxically high PTH [4]. Clinically, PHP typically presents with symptoms of hypocalcemia. However, it is important to consider other complications, such as hypokalemia or neuropsychiatric disturbances due to Fahr syndrome, which are often diagnosed years after the initial presentation [5]. The following case presents a woman with PHP associated with chronic hypocalcemia who was later found to have hypokalemia and computed tomography (CT) imaging suggestive of Fahr syndrome.

## Case Presentation

A 39-year-old woman with hypertension, obesity, tobacco use, and PHP complicated by hypocalcemia and seizure presented to the emergency department with a 4-day history of worsening headache. She reported associated symptoms of photosensitivity, eye lacrimation, perioral numbness, and infrequent muscle cramping without muscle twitching. In addition, she described a 3-month history of mild difficulty with word finding, dropping objects, and gait unsteadiness including frequent tripping. No recent falls or changes in vision. Her hypocalcemia was diagnosed at age 30 after a new-onset seizure. She was subsequently followed by an outpatient endocrinologist but was lost to follow-up after 2 years. Her home medications consisted of oral calcium carbonate 500 mg 3 times a day and bupropion 150 mg twice a day. She did not take vitamin D supplementation. She was never prescribed antiepileptic medications and had no further seizures. No surgical history. No known family history of calcium, parathyroid, or adrenal disorders.

## Diagnostic Assessment

Vital signs at presentation were notable for blood pressure of 212/135 mmHg, heart rate of 120/minute, and body mass index of 36.49 kg/m^2^. Her examination was notable for central adiposity, negative Chvostek's sign, alertness, conversational, cranial nerves intact, no extremity weakness or dysmetria, and normal deep tendon reflexes. No phenotypic expression of AHO.

**Figure 1.**
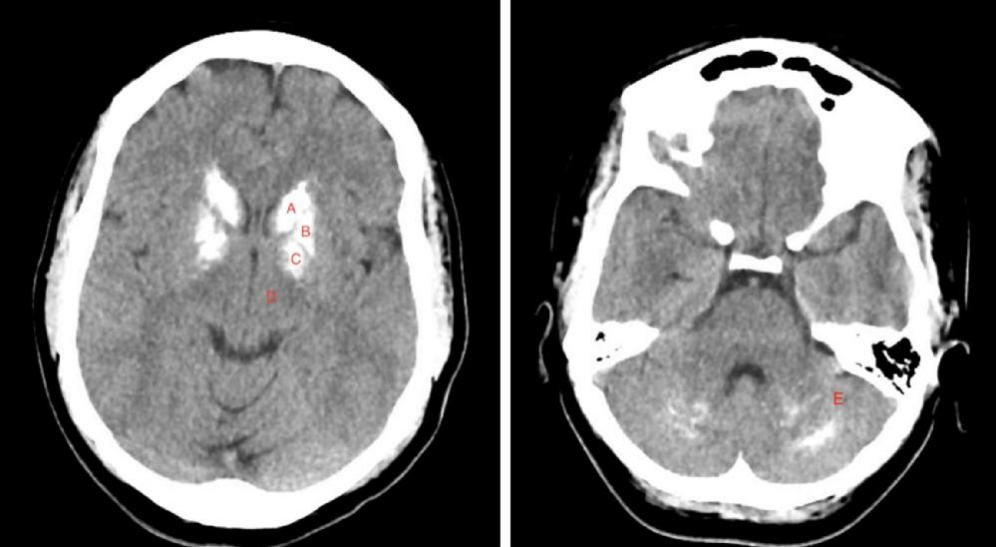
Noncontrast axial computed tomography images of the head showed calcifications in the bilateral basal ganglia, including caudate nucleus (A), putamen (B), and globus pallidus (C). The thalamus (D) appeared normal. The dentate nuclei (E) were also calcified bilaterally.

Initial investigations were consistent with PHP, noted by a low corrected calcium of 1.80 mmol/L (7.2 mg/dL, normal 8.7–10.5 mg/dL) and paradoxically elevated PTH of 383.00 ng/L (383 pg/mL, 9.0–77.0 pg/mL). Phosphorus was 1.29 mmol/L (4.0 mg/dL, 2.7–4.5 mg/dL) and albumin was 38.00 g/L (3.8 g/dL, 3.5–5.2 g/dL). Potassium was 2.9 mmol/L (2.9 mEq/L, 3.5–5.1 mEq/L) and bicarbonate was 18 mmol/L (18 mEq/L, 23–29 mEq/L). Aldosterone was 296.82 pmol/L (10.7 ng/dL, 0–16.0 ng/dL), renin 33.18 pmol/L/h (1.4 ng/mL/h, 0.2–1.6 ng/mL/h), and aldosterone/renin ratio 7.6 (0–25). Her thyrotropin (TSH) level was 2.005 mIU/L (2.005 uIU/mL, 0.400–4.000 uIU/mL). Electrocardiography demonstrated sinus tachycardia and QTc of 492 ms. CT of her head without contrast (Fig. 1) revealed extensive bilateral calcifications in basal ganglia. Specifically, involvement of the caudate nuclei and lentiform nuclei (composed of the putamen and globus pallidus) was noted, while the substantia nigra and subthalamic nuclei were spared. Thalami appeared to be normal. Additionally, bilateral calcifications in the dentate nuclei, the largest of the cerebellar nuclei, were found. This pattern was suggestive of Fahr syndrome. Imaging was negative for bleeding or masses.

## Treatment

The patient was hospitalized for the management of hypertensive emergency and severe hypocalcemia, for which endocrinology was consulted. Management of hypocalcemia consisted of intravenous calcium infusion (calcium gluconate 10 g mixed in 1000 mL NaCl at 50 mL/h), oral calcium carbonate 1 g 3 times a day with meals, oral vitamin D3 1000 IU daily, and oral calcitriol 0.25 mcg twice a day. The goal for her corrected calcium was 2.00–2.13 mmol/L (8–8.5 mg/dL), with the aim that a lower calcium goal would allow continued endogenous production of PTH and prevent hypercalciuria. Additional biochemical goals consisted of an ionized calcium of 0.23–0.25 mmol/L (0.9–1.0 mg/dL), a phosphate of 0.32–2.10 mmol/L (1.0–6.5 mg/dL), and a magnesium of 0.82 mmol/L (2.0 mg/dL).

## Outcome and Follow-Up

Symptoms of hypocalcemia resolved by the following morning and laboratory tests showed corrected calcium 2.27 mmol/L (9.1 mg/dL), phosphorus 1.55 mmol/L (4.8 mg/dL), 25-hydroxy vitamin D 44.93 nmol/L (18 ng/mL, 30–96 ng/mL), and albumin 35.00 g/L (3.5 g/dL). The calcium drip was discontinued. Her serum potassium normalized to 4.3 mmol/L (4.30 mEq/L). Laboratory values were notably negative for hyperaldosteronism. She continued to have symptoms of word-finding difficulties and gait unsteadiness. Once her blood pressure was controlled with lisinopril and amlodipine, she was discharged to home with planned primary care physician follow-up for optimization of blood pressure, endocrinology follow-up for management of hypocalcemia, and neurology follow-up for management of ongoing Fahr syndrome neurologic symptoms.

## Discussion

This case presents a woman with phenotypic PHP-1b subtype complicated by chronic hypocalcemia associated with hypokalemia and hyperphosphatemia, and further complicated by symptomatic bilateral brain calcifications consistent with Fahr syndrome. Fahr syndrome is theorized to be caused by dysregulation of serum calcium and phosphorus levels, as well as changes in the integrity of the blood-brain barrier. Once calcified, the brain tissue cannot return to its normal state [2]. Therefore, the primary management of Fahr syndrome consists of treating its root cause to prevent disease progression and providing symptomatic relief. In cases of parathyroid dysfunction, the standard treatment consists of long-term oral calcium and calcitriol repletion. Antiepileptics, antiparkinsonian drugs, and antipsychotics are considered as pertinent.

On literature review, 2 similar cases have been reported in which PHP-1b is associated with calcifications on brain imaging and hypokalemia. Huang et al describe a 27-year-old female patient with genetically confirmed sporadic PHP-1b complicated by chronic hypocalcemia and hypokalemia [5]. Her CT showed asymptomatic bilateral calcification of the globus pallidus. Her symptoms of tetany resolved following administration of oral calcium and potassium supplementation. The authors theorize that PTH-resistant patients have reduced levels of cAMP in the kidneys, and therefore decreased signaling from the Gsα/cAMP/PKA signaling pathway, which plays a major role in luminal potassium channel function. The second case, by Yang et al, reports a 13-year-old girl, also with genetically confirmed sporadic PHP-1b complicated by chronic hypocalcemia and hypokalemia [6]. CT showed multiple calcifications in the basal ganglia. These cases in addition to ours show a pattern of hypokalemia and Fahr syndrome as features associated with the PHP-1b subtype. Although the cAMP signaling pathway has been theorized to be a causative link for hypokalemia in these patients, the association between PHP-1b and hypokalemia has yet to be fully characterized in the literature. Additionally, Bitew et al reported a case of a patient with new acute seizures in the setting of severe coronavirus disease 2019 (COVID-19) infection who was found to have laboratory values and brain calcifications consistent with Fahr syndrome, suggesting the utility of serum calcium screens for patients with COVID-19 infection given that severe hypocalcemia is an endocrine emergency and may require higher level of care [7].

Limitations of this case report include the lack of genetic analysis of our patient and her family members, the lack of an admission serum 25-hydroxy vitamin D level, and the missed opportunity to include a thiazide diuretic in the patient's antihypertensive regimen as a way of mitigating her risk of hypercalciuria. However, our case, in combination with the 2 discussed cases, provides important insight into a constellation of complications potentially associated with PHP-1b. Patients with severe hypocalcemia and elevated PTH who present with new neurological symptoms despite normal general neurologic examination may warrant consideration for brain imaging to evaluate for Fahr syndrome. Further investigations are necessary to determine the prevalence of Fahr syndrome and hypokalemia in patients with PHP, explore whether they may be significantly associated with the PHP-1b subtype, better characterize the risk factors of Fahr syndrome (eg, years of exposure to hypocalcemia, poor management of underlying conditions), and ultimately inform potential new screening pathways and guidelines for these patients.

## Learning Points

- Fahr syndrome is a rare neurologic disorder characterized by calcium deposits in the basal ganglia that can present with an array of symptoms ranging from extrapyramidal to neuropsychiatric abnormalities.
- Pseudohypoparathyroidism is a rare, heterogeneous group of disorders in which target organs demonstrate resistance or unresponsiveness to PTH due to genetic mutations.
- Patients with severe hypocalcemia and elevated PTH who present with new neurological symptoms despite normal general neurologic examination may warrant consideration for brain imaging to evaluate for Fahr syndrome.
- A few case reports, including ours, report hypokalemia and Fahr syndrome as a potential constellation of complications associated with the PHP-1b subtype, but this requires further investigation.

## Contributors

All authors made individual contributions to authorship. S.W.S., S.S., Q.I., and K.R. were involved in the diagnosis and management of this patient and manuscript submission. All authors reviewed and approved the final draft.

## Data Availability

Original data generated and analyzed for this case report are included in this published article.

## Abbreviations

AHO: Albright hereditary osteodystrophy
cAMP: cyclic adenosine monophosphate
CT: computed tomography
PHP: pseudohypoparathyroidism
PTH: parathyroid hormone
